# Roles of the pro-apoptotic factors CaNma111 and CaYbh3 in apoptosis and virulence of *Candida albicans*

**DOI:** 10.1038/s41598-022-11682-y

**Published:** 2022-05-09

**Authors:** Minsik Nam, Se Hyeon Kim, Jeong-Hoon Jeong, Suyoung Kim, Jinmi Kim

**Affiliations:** Department of Microbiology and Molecular Biology, College of Bioscience and Biotechnology, Chungnam National University, 99 Daehak-ro, Yuseong-gu, Daejeon, 34134 Republic of Korea

**Keywords:** Genetics, Microbiology

## Abstract

*Candida albicans*, a commensal and opportunistic pathogen, undergoes apoptosis in response to various stimuli, including hydrogen peroxide, acetic acid, and antifungal agents. Apoptotic processes are highly conserved among mammals, plants, and fungi, but little is known about the apoptosis-regulating factors in *C. albicans.* In this study, *C. albicans* homologs of the putative apoptosis factors were identified by database screening followed by overexpression analysis. CaNma111, a homolog of the pro-apoptotic mammalian HtrA2/Omi, and CaYbh3, a homolog of BH3-only protein, yielded increased apoptotic phenotypes upon overexpression. We showed that CaNma111 and CaYbh3 functions as pro-apoptotic regulators by examining intracellular ROS accumulation, DNA end breaks (TUNEL assay), and cell survival in *Canma111*/*Canma111* and *Caybh3/Caybh3* deletion strains. We found that the protein level of CaBir1, an inhibitor-of-apoptosis (IAP) protein, was down-regulated by CaNma111. Interestingly, the *Canma111*/*Canma111* and *Caybh3/Caybh3* deletion strains showed hyperfilamentation phenotypes and increased virulence in a mouse infection model. Together, our results suggest that CaNma111 and CaYbh3 play key regulatory roles in the apoptosis and virulence of *C. albicans*.

## Introduction

Apoptosis is a form of programmed cell death, that is highly conserved in mammals, plants, and fungi, including unicellular yeasts. The pathogenic yeast *Candida albicans* exhibits typical apoptotic markers when treated with various stimuli, including hydrogen peroxide (H_2_O_2_), acetic acid, and UV irradiation^1–3^. In addition, antifungal agents, such as amphotericin B and the quorum-sensing molecule, farnesol, can induce apoptosis^4,5^. The metacaspase CaMca1 is a caspase-related protease in *C. albicans*, that shows homology to the yeast *Saccharomyces cerevisiae* metacaspase, Yca1^6–9^. Metacaspases are known to be involved in the stress-induced cell death of the yeasts, *S. cerevisiae* and *C. albicans,* the plant, *Arabidopsis thaliana*, the fungal species, *Aspergillus nidulans,* the protozoa, *Leishmania major*^2,6,8,10^. Metacaspases are distinguished from mammalian caspases by various biochemical features, including their proteolytic processing ability and/or Arg/Lys substrate specificity.

In mammals, the activation or regulation of caspases requires various pro- and anti-apoptotic proteins, including the Bcl-2 (B-cell lymphoma) family members (Bax, Bak, Bcl-2, and Bcl-xL) and the inhibitor-of-apoptosis proteins (IAPs)^11–13^. *S. cerevisiae* appears to lack homologs of the Bcl-2 proteins with the exception of the yeast BH3-only protein, Ybh3^13,14^. A few other apoptotic regulators have been identified in *S. cerevisiae*, including the single IAP (inhibitor-of-apoptosis), Bir1, the pro-apoptotic protease, Omi/HtrA2 (Nma111) and an apoptosis-inducing factor (Aif1)^14–16^. In *C. albicans,* little is known about the regulation of apoptosis or metacaspase activation*.* Recently, a single IAP, CaBir1, was identified to inhibit apoptosis by lowering intracellular caspase-like activity in *C. albicans* ^17–19^. The *Cabir1/Cabir1* deletion mutant showed increased apoptotic features, including ROS accumulation and nuclear segmentation.

To investigate the regulatory mechanisms underlying apoptotic processes in *C. albicans*, we searched for putative pro-apoptotic or anti-apoptotic regulators by employing an overexpression strategy. Among five candidates screened, CaNma111 and CaYbh3 exhibited pro-apoptotic activity and were further characterized by constructing the deletion mutant strains. We also showed hyperfilamentation phenotypes and increased virulence of the *Canma111*/*Canma111* and *Caybh3/Caybh3* deletion strains.

## Results

### Overexpression of putative apoptosis factors in *C. albicans*

**Figure 1:**
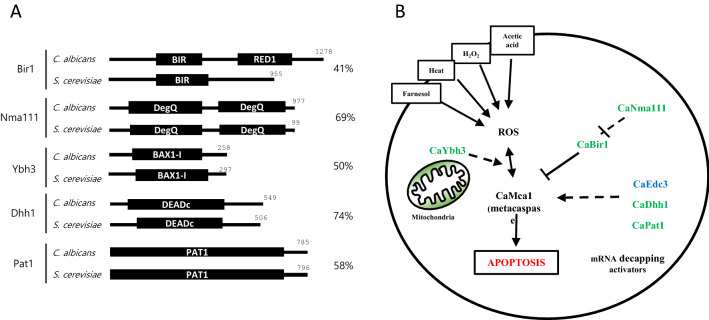
Schematic representation of putative apoptotic factors, CaBir1, CaNma111, CaYbh3, CaDhh1, and CaPat1 in *C. albicans* (**A**) The alignments of the respective protein sequences from *C. albicans* and *S. cerevisiae* are presented. The presence of putative conserved domains is indicated with shaded boxes. The number at the end of each protein represents the total amino acid length. Percentage indicates similarity of each *C. albicans* protein with its homolog in *S. cerevisiae.* (**B**) Model for putative apoptotic factors and pathways of *C. albicans*. *C. albicans* orthologs of apoptosis-regulating factors are indicated in yeast apoptotic pathway. Predicted pathway locations of CaBir1, CaNma111, CaYbh3, CaDhh1, and CaPat1 are presented with the dashed arrows. Roles of each protein are predicted from those of the corresponding orthologs in *S. cerevisiae* and mammals.

To investigate putative apoptosis-regulating factors in *C. albicans*, we constructed overexpression strains of five genes: *CaBIR1, CaNMA111, CaYBH3, CaDHH1* and *CaPAT1* (Fig. 1). These genes were identified from the Candida Genome Database based on their amino acid sequence similarities with homologous proteins in *S. cerevisiae* and mammals. CaBir1, a single IAP in *C. albicans*, was shown to inhibit apoptosis by reducing caspase-like activity under an oxidative stress condition^19^*.* CaNma111 (nuclear mediator of apoptosis) is a homolog of the pro-apoptotic serine protease, HtrA2/Omi. In mammals and *S. cerevisiae*, HtrA2/Omi regulates apoptosis by binding and degrading cellular IAPs^15,16^. Ybh3 is the yeast homolog of the BH3-only protein, which contains a BCL-2 homology domain (BH3)^13,14^. We identified its homolog, CaYbh3, in *C. albicans.* Dhh1 and Pat1, which are known as the components of P-bodies (processing bodies, mRNA granules) in *S. cerevisiae*, function as mRNA-decapping activators^20,21^. CaDhh1, which was identified in a previous work, was shown to be localized to P-bodies in *C. albicans*^22^. CaPat1 was identified as *C. albicans* homolog in this study.

For ectopic overexpression, each target gene was cloned downstream of the *ACT1* promoter in plasmid pPR671, and the constructed plasmid was chromosome-integrated into the wild-type *C. albicans* strain^23^.

### Overexpression of CaNma111 or CaYbh3 yields increased apoptotic phenotypes

**Figure 2:**
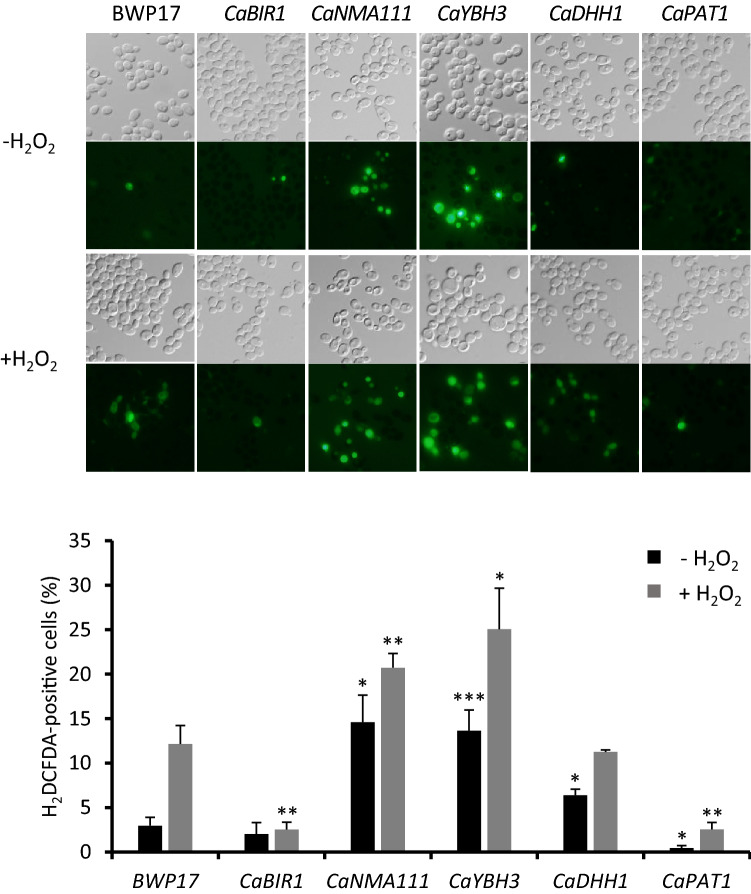
ROS accumulation was measured in cells overexpressing CaBir1, CaNma111, CaYbh3, CaDhh1, or CaPat1. The wild-type BWP17 and overexpression strains were grown to early log phase, incubated with 50 μg/ml H_2_DCFDA for 60 min, and then treated with 10 mM H_2_O_2_. ROS-stained cells were observed under an Olympus BX51 microscope with a 60 × objective. Graphs represent the quantification of ROS-stained cells (%) (n = 3 replicates, > 200 cells). Values are presented as the mean ± SD; * *p* < 0.05, ** *p* < 0.01, *** *p* < 0.005 (compared with the wild-type BWP17).

Apoptosis is characterized by several morphological and biochemical features including chromatin condensation, accumulation of reactive oxygen species (ROS), and increased caspase activity^6,24^. In each overexpression strain, we determined the intracellular amount of ROS by staining cells with the fluorescent dye, H_2_DCFDA (Fig. 2). Compared to wild-type BWP17 cells, overexpression strains OE*CaNMA111* and OE*CaYBH3* showed increased frequencies of H_2_DCFDA-positive cells before and after apoptosis-inducing H_2_O_2_ treatment. OE*CaBIR1* and OE*CaPAT1* showed decreased ROS accumulation compared to the wild-type. OE*CaDHH1* showed a ROS level higher than that of wild-type cells prior to H_2_O_2_ treatment but similar to that of wild-type cells after H_2_O_2_ treatment.

**Figure 3:**
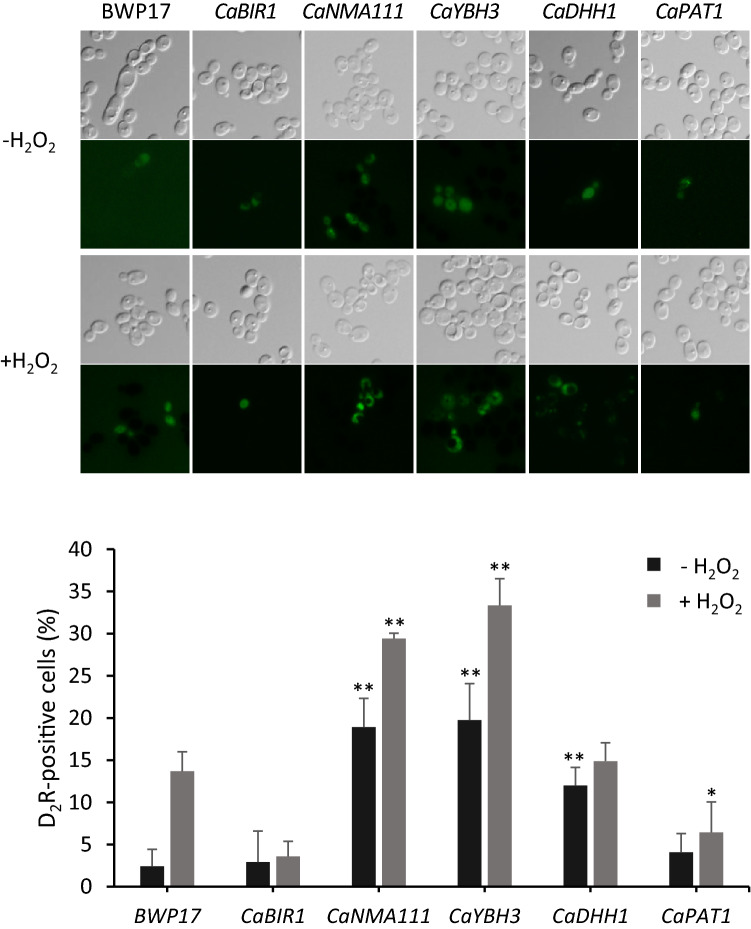
Caspase-like activities were measured in cells overexpressing CaBir1, CaNma111, CaYbh3, CaDhh1, or CaPat1. The wild-type and overexpression strains were grown to early log phase and treated with 10 mM H_2_O_2_ for 30 min. Cells (5 × 10^6^) were washed with PBS and incubated for 20 min in the presence of D_2_R. The D_2_R-stained cells were observed under an Olympus BX51 microscope with a 60 × objective. Graphs represent the quantification of D_2_R -stained cells (%) (n = 3 replicates, > 200 cells). Values are mean ± SD. * *p* < 0.05, ** *p* < 0.01 (compared with the wild-type BWP17).

Next, we measured the caspase-like activity in the overexpression strains using the caspase substrate, D_2_R (Asp_2_Rhodamine 110). Cells were stained with D_2_R and the frequency of fluorescent D_2_R-positive cells was calculated (Fig. 3). In the wild-type strain, BWP17, few cells were fluorescent in H_2_O_2_-untreated cells, but numerous fluorescent cells were observed after 30 min of H_2_O_2_ stress. OE*CaNMA111* and OE*CaYBH3* showed increases in the frequency of fluorescent cells compared to the wild-type strain with or without oxidative stress. In contrast, OE*CaBIR1* and OE*CaPAT1* showed little fluorescence, regardless of H_2_O_2_ treatment. OE*CaDHH1* showed an increase in the number of fluorescent cells before H_2_O_2_ treatment, but only a slight increase after H_2_O_2_ treatment. These patterns of caspase-like activity in each overexpression strain were closely associated with the ROS accumulation level.

### Deletion of *CaNMA111* or *CaYBH3* decreased apoptotic phenotypes

**Figure 4:**
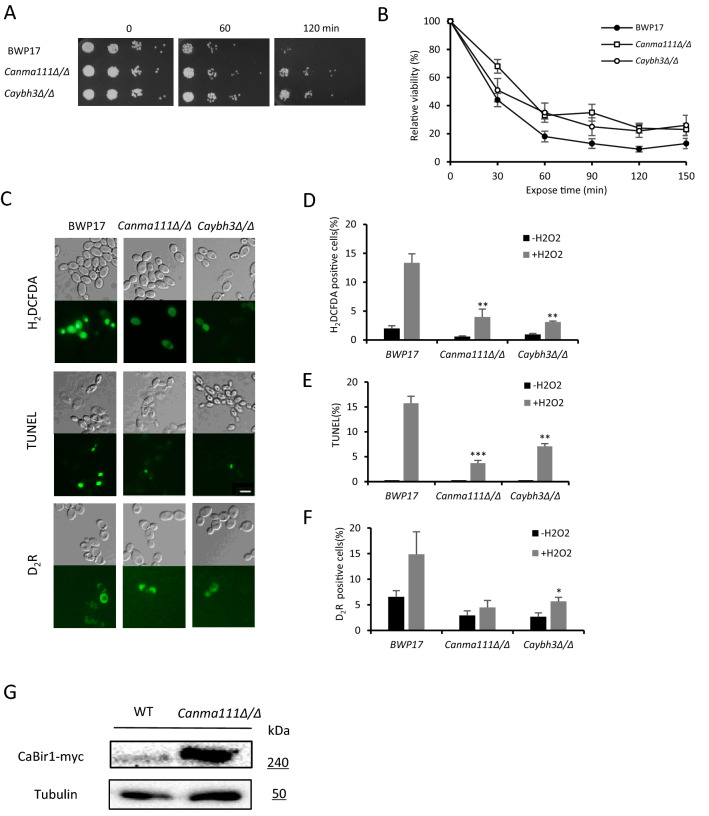
The apoptotic phenotypes of *Canma111/Canma111* and *Caybh3/Caybh3* mutant strains. (**A**) Survival of the wild-type, *Canma111/Canma111*, and *Caybh3/Caybh3* strains was examined by spot assays. The cells were grown to early log phase and treated with 7.5 mM H_2_O_2_ for 2 h. Serial dilutions of cells were spotted onto YEPD plates, which were incubated at 30 °C and photographed after 2 days. (**B**) Relative viabilities of the wild-type, *Canma111/Canma111*, and *Caybh3/Caybh3* strains were determined upon H_2_O_2_ treatment. Cells in early log phase were treated with 5 mM H_2_O_2_ for the indicated time. Culture samples were diluted and plated in duplicate. Viabilities were scored as a percentage of the number of colonies formed at time zero. (**C**) Fluorescence microscopy of ROS, TUNEL, or D_2_R staining of the wild-type, *Canma111/Canma111*, and *Caybh3/Caybh3* strains following treatment with H_2_O_2_. Cells were observed under an Olympus BX51 microscope with a 60 × objective. Scale bar, 5 μm. (**D**) Graphs represent the quantification of ROS-stained cells (%) (n = 3 replicates, > 200 cells). Values are presented as the mean ± SD; ** *p* < 0.01. (**E**) Quantification of TUNEL-positive cells are graphed (n = 3 replicates, > 200 cells). TUNEL assays were carried out after cells were exposed to 7.5 mM H_2_O_2_ for 2.5 h. The percentages of values are presented as mean ± SD; ** *p* < 0.01, *** *p* < 0.005. (**F**) Graphs represent the quantification of D_2_R -stained cells (%) (n = 3 replicates, > 200 cells). Cells were treated with 10 mM H_2_O_2_ for 30 min. Values are mean ± SD. * *p* < 0.05. (**G**) Detection of the CaBir1-myc protein band in the *Canma111/Canma111* mutant strain. The PR671-derived *ACT1-CaBIR1-MYC* construct was chromosomally integrated in the wild-type and *Canma111/Canma111* mutant strains. Western blotting was conducted using anti-myc antibody. Tubulin was detected as a loading control.

To further investigate the roles of *CaNMA111* and *CaYBH3* in apoptosis, we sequentially deleted the two copies of *CaNMA111* or *CaYBH3* to construct the *Canma111/Canma111* and *Caybh3/Caybh3* deletion strains, respectively. The wild-type and deletion mutant strains were compared for apoptotic hallmarks, including ROS accumulation, nuclear segmentation (TUNEL assay), and cell survival under oxidative stress (Fig. 4). Cell survival after H_2_O_2_ treatment was much higher in *Canma111/Canma111* and *Caybh3/Caybh3* mutant cells than wild-type cells (Fig. 4A, B). The amount of ROS, which was determined using the fluorescent dye, H_2_DCFDA, was lower in *Canma111/Canma111* and *Caybh3/Caybh3* cells than wild-type cells upon H_2_O_2_ treatment. The TUNEL assay, which measures DNA breaks, revealed that *Canma111/Canma111* and *Caybh3/Caybh3* mutant cells showed lower frequencies of TUNEL-positive nuclei than wild-type cells upon H_2_O_2_ treatment. The mutant strains showed decreased caspase-like activity compared to wild-type cells following treatment with H_2_O_2_ for 30 min (Fig. 4C, F). These results collectively suggest that CaNma111 and CaYbh3 are required for apoptotic cell death in *C. albicans*.

### CaNma111 downregulates the apoptosis inhibitor, CaBir1

The mammalian serine protease, Omi/HtrA2, promotes apoptosis by binding and degrading IAP family proteins^25,26^. Consistently, the yeast IAP, Bir1, was shown to be a substrate for Nma111 in *S. cerevisiae*^15^. We repeatedly observed a very faint protein band when we assessed chromosome-tagged CaBir1-GFP or CaBir1-myc in a wild-type background (data not shown). To ask whether CaNma111 is one of the proteases responsible for the degradation of CaBir1, we compared CaBir1-myc levels in wild-type and *Canma111/Canma111* cells. Here, CaBir1-myc was expressed under the control of the *ACT1* promoter of the pPR671 vector. We observed an increased level of CaBir1-myc in *Canma111/Canma111* cells, compared to wild-type cells (Fig. 4G). This result suggests that CaNma111 downregulates CaBir1 in *C. albicans*.

### Deletion of *CaNMA111* or significantly increases filamentous growth and virulence

*C. albicans* is an opportunistic pathogen and switches rapidly among the budding yeast, pseudohyphal, and hyphal forms in response to environmental changes^27,28^. This morphogenetic switching is particularly associated with virulence. In addition, it has been suggested that the morphological state affects apoptotic cell death^29^.

**Figure 5:**
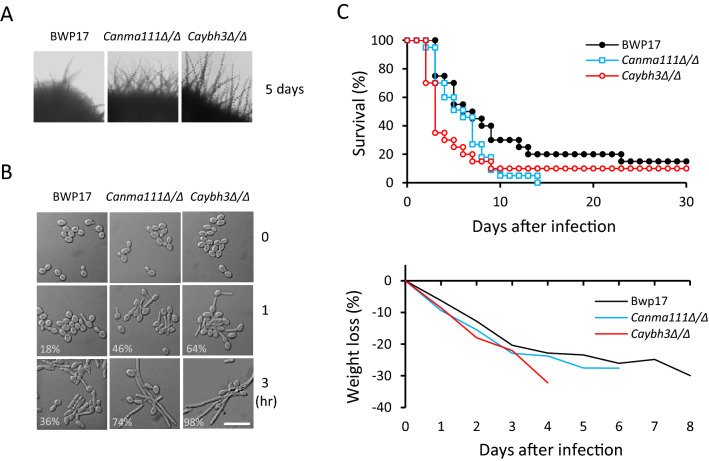
The hyphal phenotype and virulence of *Canma111/Canma111* and *Caybh3/Caybh3* mutant strains. (**A**) Colony morphologies of wild-type (BWP17 + pRC18), *Canma111/Canma111*, and *Caybh3/Caybh3* cells grown on Spider medium at 37 °C for 5 days and photographed at 100 × magnification. (**B**) Cell morphologies of wild-type (BWP17 + pRC18), *Canma111/Canma111*, and *Caybh3/Caybh3* cells grown in serum-containing medium at 37 °C and photographed at 600 × magnification. The percentage of cells with a germ-tube (1 h) or a filament (3 h) was analyzed for 100 cells each. (**C**) Survival and weight loss of BALB/c mice after inoculation with the wild-type (BWP17 + pRC18), *Canma111/Canma111*, and *Caybh3/Caybh3* strains. Each strain (6 × 10^5^ CFU) was injected into 10 mice, and host survival was monitored for 30 days. Survival curve represents the average of two independent infection experiments. Weight loss was monitored for 8 days and the curve represents the average of 10 mice.

We therefore examined whether the pro-apoptotic regulators, CaNma111 and CaYbh3, are involved in the filamentous growth or virulence of *C. albicans*. Colony morphologies of the wild-type, *Canma111/Canma111*, and *Caybh3/Caybh3* strains were examined on hyphae-inducing solid medium. As shown in Fig. 5A, *Canma111/Canma111* and *Caybh3/Caybh3* mutant cells exhibited hyperfilamentation phenotypes on solid Spider medium, compared with wild-type cells. Interestingly, the mutant strains showed filamentous growth on YEPD complete medium (data not shown). The hyperfilamentation phenotypes of *Canma111/Canma111* and *Caybh3/Caybh3* mutant strains were also evident in liquid medium supplemented with 10% serum (Fig. 5B).

The virulence of the *Canma111/Canma111* and *Caybh3/Caybh3* strains was tested in a tail vein-infection model with BALB/c mice. Two groups of mice (n = 10) were challenged with wild-type and mutant cells and survival was monitored for up to 30 days. Mice infected with *Canma111/Canma111* or *Caybh3/Caybh3* mutant strain showed more rapid weight loss and decreased survival than those infected with wild-type BWP17 (Fig. 5C). These results demonstrate that *CaNMA111* and *CaYBH3* play important roles in the virulence of *C. albicans*.

## Discussion

We analyzed five candidate regulators for apoptosis in *C. albicans* and found that overexpression of CaNma111 or CaYbh3 yielded pro-apoptotic features, while that of CaBir1 or CaPat1 yielded anti-apoptotic feature. CaNma111 and CaYbh3 were further characterized by constructing the deletion mutant strains. Gene overexpression mimics gain-of-function mutations, and thus offers a useful approach for revealing pathways or pathway components in the diploid pathogen, *C. albicans*^30–32^. The overexpression phenotypes of the apoptosis inhibitor, CaBir1, were consistent with our recent report that the *Cabir1/Cabir1* mutant strain showed increased apoptotic phenotypes, such as ROS accumulation and DNA fragmentation, under apoptosis-inducing conditions^19^. We analyzed CaDhh1 and CaPat1 because our previous work showed that CaEdc3, another component of P-bodies, is involved in apoptosis^33^. CaEdc3 contributes to the expression of CaMca1 expression and thereby functions as a pro-apoptotic factor. In our overexpression analysis, CaPat1 was suggested to be anti-apoptotic factor. We suggest that CaDhh1, CaEdc3, and CaPat1 could all participate in apoptosis, with each playing a distinct role. CaDhh1 and CaPat1 show protein interactions with each other but differ in their functional domains, intracellular locations, and mRNA targets^20,34^.

Here, we report our results from the deletion mutant analysis of *CaNMA111* and *CaYBH3*. The decreased apoptotic phenotypes of *Canma111/Canma111* and *Caybh3/Caybh3* mutant cells suggest that CaNma111 and CaYbh3 function as pro-apoptotic regulators in *C. albicans*. HtrA2/Omi, which is a mammalian counterpart of CaNma111, has been identified as a direct IAP-binding protein^16,26^. It exerts pro-apoptotic character effects, possibly by disruption of the IAP-caspase interaction. Studies have shown that the serine protease, HtrA2/Omi, can degrade mammalian IAP and XIAP^25,26^. We repeatedly observed very faint protein band corresponding to CaBir1-GFP or CaBir1-myc in a wild-type background (data not shown). We speculated that the full-length CaBir1 protein could be a target of proteolytic degradation. Our observation that the CaBir1-myc protein level was increased in *Canma111/Canma111* cells may support this notion. However, future work is needed to assess whether CaNma111 could be among the proteases responsible for CaBir1 degradation.

The hyperfilamentation phenotypes and increased virulence of the *Canma111/Canma111* and *Caybh3/Caybh3* mutant strains were particularly interesting, as these findings suggest that the pro-apoptotic regulators, CaNma111 and CaYbh3, exert repressive actions on filamentation and pathogenicity in *C. albicans*. It remains unknown whether the pro-apoptotic roles of CaNma111 and CaYbh3 overlap with their functions during morphogenesis. One possible explanation is that CaNma111, which is a serine protease responsible for degrading the apoptosis inhibitor, CaBir1, could be involved in the processing or breakdown of regulatory factors crucial for filamentous growth. Further studies will be needed to uncover the downstream targets of CaNma111 or CaYbh3 protease activity during morphogenesis. Regarding CaYbh3, we speculate that a putative BH3 domain within this *C. albicans* protein could be responsible for mitochondria-driven ROS accumulation and/or the release of apoptotic factors^13^. During hyphal morphogenesis, *C. albicans* produces a burst of ROS that is mainly located at the hyphal tip^35,36^. Further investigation is needed to examine whether the repressive function of CaYbh3 during filamentous growth could also be associated with changes in the ROS level.

It is noteworthy that the pro-apoptotic regulator, CaMca1 metacaspase, was shown to be required for filamentation and pathogenicity^37^. Cells harboring the apoptosis-defective deletion of *CaMCA1* or the catalytic-site mutation *CaMCA1*^*c292*^, showed defects in filamentation and virulence. It has been suggested that *S. cerevisiae* metacaspases, which are responsible for apoptosis, are also involved in nonapoptotic characteristics and processes, such as longevity, the fitness of growing cells, and protein clearance^38,39^. We speculate that the downstream targets of CaNma111 protease and CaMca1 metacaspase could act to either promote or repress filamentous growth and other nonapoptotic processes.

Various regulatory elements involved in the yeast-to-hyphal transition has been identified in *C. albicans*^27,40,41^. The Ras-cAMP-PKA and the MAPK pathway operate to promote the yeast-to-hyphal transition and the transcription factors, such as Cph1 and Efg1, are targets of these pathway responses in *C. albicans*. Activation of Ras-signaling was shown to accelerate apoptotic responses under treatment with acetic acid or H_2_O_2_^42^. However, little is known about the interrelationship between cell death and morphogenesis. The quorum-sensing molecule, farnesol, inhibits the yeast-to-hyphal switch, but this triggers apoptosis^4,5^. Going forward, additional work is needed to improve our understanding of the detailed regulatory points and components involved in the apoptotic responses and pathogenicity of *C. albicans.*

## Materials and methods

### Strains, plasmids, and culture conditions

The *C. albicans* strains and plasmids used in this study are listed in Table S1. Constructions of the *Canma111/Canma111* and *Caybh3/Caybh3* deletion strains were essentially as described previously^22,40^. We used plasmids pJI434 and pJI435 for *CaNMA111* deletion, and plasmids pJI436 and pJI437 for *CaYBH3* deletion. These plasmids carried the deletion cassettes, *hph-URA3-hph* and *hisG-URA3-hisG*, respectively. Each disruption was verified by PCR. Overexpression strains were constructed using the pPR671-derived plasmids, pJI426-pJI432. Each target gene was amplified using a primer set (Table S2), and the PCR fragment was digested with *Mlu*I/*Xma*I and ligated into the *Mlu*I and *Xma*I sites of pPR671^22^. Each pPR671-derived plasmid was linearized by *Stu*I and transformed into the wild-type BWP17 strain. Chromosome integrations were verified by PCR and protein expressions were analyzed by Western blot.

*C. albicans* strains were cultured in YEPD (1% yeast extract, 2% peptone, 2% dextrose) or SC (synthetic complete; 0.67% yeast nitrogen base w/o amino acid, 2% glucose, all required amino acids) medium. The filamentation phenotype of *C. albicans* cells was tested in serum-containing medium (YEPD with 10% new born calf serum) and Spider medium (1% mannitol, 1% nutrient broth, 0.2% K_2_HPO_4_, pH7.2) as described previously^43^.

### ROS accumulation assay

Early exponential cells were pre-incubated with 50 μg/ml of 2’,7’– dichlorofluorescin diacetate (H_2_DCFDA, Sigma-Aldrich, USA) for 1 h. Cells were treated with 10 mM H_2_O_2_ for 1 h or 2 h, washed with PBS, and observed under an Olympus BX51 microscope equipped with a 60 × objective.

### Caspase assay

Caspase activity was analyzed using a CaspSCREEN™ flow cytometric apoptosis detection kit (BioVision, USA). Early exponential cells were incubated with 10 mM H_2_O_2_ for 30 min at 30 ^o^C, and then washed with PBS, and suspended in D_2_R (aspartyl)_2_-rhodamine) reagent for 30 min. Cells were washed with PBS and observed under an Olympus BX51 microscope equipped with a 60 × objective.

### Cell survival assay

Early exponential cells were treated with 5 mM H_2_O_2_ at 30 °C. Cells were collected every 30 min, diluted in PBS, and plated to YEPD plates. Colonies were counted after a 2-day incubation at 30 °C.

### TUNEL assay

DNA strand breaks were demonstrated by TUNEL (TdT-mediated dUTP nick end labeling) assay using an In Situ Cell Death Detection kit (Roche Molecular Biochemicals, Germany), as described previously^44^. Yeast cells were fixed with 3.7% formaldehyde, digested with 12 μg/ml zymolyase 100 T (10^6^ units/g; US Biological, USA) at 30 °C for 45 min, and applied to a poly-lysine-coated slide. Each slide was rinsed with PBS and incubated in permeabilization solution (0.1% Triton X-100, 0.1% sodium citrate) for 2 min on ice. The slides were incubated with a TUNEL reaction mixture containing terminal deoxynucleotidyl transferase and FITC-labeled dUTP, and mounted with a drop of VECTASHIELD antifading agent (Vector Laboratories Inc., USA). Observations were made with an Olympus BX51 microscope equipped with a 60 × objective.

### Western blot analysis

Total protein preparation and Western blotting were performed as previously described^22^. Myc-tagged proteins were detected with anti-myc antibody (Roche, USA). HRP-conjugated anti-mouse IgG antibody (Santa Cruz Biotechnology, USA) was used as the secondary antibody. Tubulin protein was used as a loading control, and was detected with a monoclonal anti-α-tubulin antibody (Sigma-Aldrich, USA). Protein bands were visualized using an Enhanced Peroxidase Detection (EPD) Western reagent kit (Elpis-Biotech, KR).

### Assessment of virulence in a murine infection model

Cells were grown overnight in SC-Ura medium and washed twice with sterile physiological saline. Seven-week-old female BALB/c mice were infected via lateral tail vein injection with 6 × 10^5^ CFU (colony forming unit) in a 100-μl volume. Ten mice were inoculated per test strain, and host survival was monitored over 30 days. All animal experiments were approved by the Animal Experiment Ethics Committee of Chungnam National University (approval No. 202006A-CNU-120, July 2020) and performed in accordance with the guidelines of the Ethics Training Guidelines for Experiments on Animals of CNU Animal Research Center. This study additionally adheres to standards articulated in the ARRIVE guidelines.

## Supplementary Information

Supplementary Information 1.

Supplementary Information 2.

## References

[1] Phillips AJ, Sudbery I, Ramsdale M (2003). Apoptosis induced by environmental stresses and amphotericin B in *Candida albicans*. Proc. Natl. Acad. Sci. U S A.

[2] Cao Y (2009). *Candida albicans* cells lacking *CaMCA1*-encoded metacaspase show resistance to oxidative stress-induced death and change in energy metabolism. Fungal Genet. Biol..

[3] Lin SJ, Austriaco N (2014). Aging and cell death in the other yeasts, *Schizosaccharomyces pombe* and *Candida albicans*. FEMS Yeast Res..

[4] Shirtliff ME (2009). Farnesol-induced apoptosis in *Candida albicans*. Antimicrob. Agents Chemother..

[5] Leger T, Garcia C, Ounissi M, Lelandais G, Camadro JM (2015). The metacaspase (Mca1p) has a dual role in farnesol-induced apoptosis in *Candida albicans*. Mol. Cell. Proteomics.

[6] Madeo F (2002). A caspase-related protease regulates apoptosis in yeast. Mol. Cell..

[7] Vercammen D, Declercq W, Vandenabeele P, Van Breusegem F (2007). Are metacaspases caspases?. J. Cell. Biol..

[8] Tsiatsiani L (2011). Metacaspases. Cell Death Differ..

[9] Wong AH, Yan C, Shi Y (2012). Crystal structure of the yeast metacaspase Yca1. J. Biol. Chem..

[10] He R (2008). Metacaspase-8 modulates programmed cell death induced by ultraviolet light and H_2_O_2_ in Arabidopsis. J. Biol. Chem..

[11] Youle RJ, Strasser A (2008). The BCL-2 protein family: opposing activities that mediate cell death. Nat. Rev. Mol. Cell Biol..

[12] Renault TT, Dejean LM, Manon S (2017). A brewing understanding of the regulation of Bax function by Bcl-xL and Bcl-2. Mech. Ageing Dev..

[13] Buttner S (2011). A yeast BH3-only protein mediates the mitochondrial pathway of apoptosis. EMBO J..

[14] Polcic P, Jaka P, Mentel M (2015). Yeast as a tool for studying proteins of the Bcl-2 family. Microb. Cell.

[15] Walter D, Wissing S, Madeo F, Fahrenkrog B (2006). The inhibitor-of-apoptosis protein Bir1p protects against apoptosis in *S. cerevisiae* and is a substrate for the yeast homologue of Omi/HtrA2. J. Cell Sci..

[16] Fahrenkrog B, Sauder U, Aebi U (2004). The *S-cerevisiae* HtrA-like protein Nma111p is a nuclear serine protease that mediates yeast apoptosis. J. Cell Sci..

[17] Uren AG (1999). Role for yeast inhibitor of apoptosis (IAP)-like proteins in cell division. Proc. Natl. Acad. Sci. USA.

[18] O'Riordan MX, Bauler LD, Scott FL, Duckett CS (2008). Inhibitor of apoptosis proteins in eukaryotic evolution and development: a model of thematic conservation. Dev. Cell.

[19] Jeong JH, Kim SH, Kim J (2021). CaBir1 functions as an inhibitor-of-apoptosis and affects caspase-like activitiy in *Candida albicans*. Fungal Genet. Biol..

[20] Pilkington GR, Parker R (2008). Pat1 contains distinct functional domains that promote P-body assembly and activation of decapping. Mol. Cell Biol..

[21] Nissan T, Rajyaguru P, She M, Song H, Parker R (2010). Decapping activators in *Saccharomyces cerevisiae* act by multiple mechanisms. Mol. Cell.

[22] Jung JH, Kim J (2011). Accumulation of P-bodies in *Candida albicans* under different stress and filamentous growth conditions. Fungal Genet. Biol..

[23] Cao F (2006). The Flo8 transcription factor is essential for hyphal development and virulence in *Candida albicans*. Mol. Biol. Cell.

[24] Carmona-Gutierrez D (2010). Apoptosis in yeast: triggers, pathways, subroutines. Cell Death Differ..

[25] Srinivasula SM (2003). Inhibitor of apoptosis proteins are substrates for the mitochondrial serine protease Omi/HtrA2. J. Biol. Chem..

[26] Hegde R (2002). Identification of Omi/HtrA2 as a mitochondrial apoptotic serine protease that disrupts inhibitor of apoptosis protein-caspase interaction. J. Biol. Chem..

[27] Calderone RA, Fonzi WA (2001). Virulence factors of *Candida albicans*. Trends Microbiol..

[28] Sudbery P, Gow N, Berman J (2004). The distinct morphogenic states of *Candida albicans*. Trends Microbiol..

[29] Laprade DJ, Brown MS, McCarthy ML, Ritch JJ, Austriaco N (2016). Filamentation protects *Candida albicans* from amphotericin B-induced programmed cell death via a mechanism involving the yeast metacaspase, MCA1. Microb. Cell.

[30] Cabral V (2014). Targeted changes of the cell wall proteome influence *Candida albicans* ability to form single- and multi-strain biofilms. PLoS Pathog..

[31] Prelich G (2012). Gene overexpression: uses, mechanisms, and interpretation. Genetics.

[32] Znaidi S (2018). Systematic gene overexpression in *Candida albicans* identifies a regulator of early adaptation to the mammalian gut. Cell Microbiol..

[33] Jung JH, Kim J (2014). Roles of Edc3 in the oxidative stress response and *CaMCA1*-encoded metacaspase expression in *Candida albicans*. FEBS J..

[34] Vindry C, Weil D, Standart N (2019). Pat1 RNA-binding proteins: multitasking shuttling proteins. Wiley Interdiscip. Rev. RNA.

[35] Kowalewski GP (2021). Cdc42 regulates reactive oxygen species production in the pathogenic yeast *Candida albicans*. J. Biol. Chem..

[36] Rossi DCP (2017). *Candida albicans FRE8* encodes a member of the NADPH oxidase family that produces a burst of ROS during fungal morphogenesis. PLoS Pathog..

[37] Jeong JH, Lee SE, Kim J (2016). Mutational analysis of metacaspase CaMca1 and decapping activator Edc3 in the pathogenicity of *Candida albicans*. Fungal Genet. Biol..

[38] Hill SM, Nystrom T (2015). The dual role of a yeast metacaspase: what doesn't kill you makes you stronger. BioEssays.

[39] Lee RE, Brunette S, Puente LG, Megeney LA (2010). Metacaspase Yca1 is required for clearance of insoluble protein aggregates. Proc. Natl. Acad. Sci. USA.

[40] Feng Q, Summers E, Guo B, Fink G (1999). Ras signaling is required for serum-induced hyphal differentiation in *Candida albicans*. J. Bacteriol.

[41] Sudbery PE (2011). Growth of *Candida albicans* hyphae. Nat. Rev. Microbiol..

[42] Phillips AJ, Crowe JD, Ramsdale M (2006). Ras pathway signaling accelerates programmed cell death in the pathogenic fungus *Candida albicans*. Proc. Natl. Acad. Sci. USA.

[43] Liu H, Kohler J, Fink GR (1994). Suppression of hyphal formation in *Candida albicans* by mutation of a *STE12* homolog. Science.

[44] Madeo F (1999). Oxygen stress: a regulator of apoptosis in yeast. J. Cell Biol..

