# Supplementary material for: Roles of the pro-apoptotic factors CaNma111 and CaYbh3 in apoptosis and virulence of Candida albicans
Source: Sci Rep. 2022 May 9;12:7574. doi: 10.1038/s41598-022-11682-y (PMC9085738; doi:10.1038/s41598-022-11682-y)
Supplement: Supplementary file 1 — Supplementary Information 1. [file 41598_2022_11682_MOESM1_ESM.pdf]

Roles of the pro-apoptotic factors CaNma111 and CaYbh3 in apoptosis and virulence of *Candida albicans*

*Minsik Nam[1,2], Se Hyeon Kim[1,2], Jeong-Hoon Jeong[1], Su Young Kim[1], and Jinmi Kim[1]**

Table S1. Strains and plasmids used in this study

Strains	Genotype	Reference
BWP17	*ura3::imm434/ura3::imm434 his1::hisG/his1::hisG arg4::hisG/arg4::hisG*	1
JKC136	*ura3::imm434/ura3::imm434 his1::hisG/his1::hisG arg4::hisG/arg4::hisG, ACT1$_P$-DHH1::13MYC-FLAG, HIS1*	In this study
JKC137	*ura3::imm434/ura3::imm434 his1::hisG/his1::hisG arg4::hisG/arg4::hisG, ACT1$_P$-PAT1::13MYC-FLAG HIS1*	In this study
JKC138	*ura3::imm434/ura3::imm434 his1::hisG/his1::hisG arg4::hisG/arg4::hisG, ACT1$_P$- YBH3::13MYC-FLAG HIS1*	In this study
JKC139	*ura3::imm434/ura3::imm434 his1::hisG/his1::hisG arg4::hisG/arg4::hisG, ACT1$_P$-NMA111::13MYC-FLAG HIS1*	In this study
JKC140	*ura3::imm434/ura3::imm434 his1::hisG/his1::hisG arg4::hisG/arg4::hisG, ACT1$_P$-BIR1::13MYC-FLAG HIS1*	In this study
JKC141	*ura3::imm434/ura3::imm434 his1::hisG/his1::hisG arg4::hisG/arg4::hisG, nma111::hph/nma111-hisG-URA3-hisG*	In this study
JKC142	*ura3::imm434/ura3::imm434 his1::hisG/his1::hisG arg4::hisG/arg4::hisG, ybh3::hph/ybh3-hisG-URA3-hisG*	In this study
JKC143	*ura3::imm434/ura3::imm434 his1::hisG/his1::hisG arg4::hisG/arg4::hisG, nma111::hph/nma111-hisG-URA3-hisG, ACT1$_P$-BIR1-13MYC-FLAG, HIS1*	In this study
**Plasmids**	**Description**	**Reference**
pPR671	*C. albicans ACT1 promoter, 13MYC-FLAG, HIS1*	2

pRC18	*URA3*-marked *CaARS* vector, containing pUC18 multiple cloning site	3
pJI426	pPR671-drived *Ori amp*^{r} *HIS1 CaBIR1::13MYC-FLAG*	In this study
pJI429	pPR671-drived *Ori amp*^{r} *HIS1 CaDHH1::13MYC-FLAG*	In this study
pJI430	pPR671-drived *Ori amp*^{r} *HIS1 CaPAT1::13MYC-FLAG*	In this study
pJI431	pPR671-drived *Ori amp*^{r} *HIS1 CaYBH3::13MYC-FLAG*	In this study
pJI432	pPR671-drived *Ori amp*^{r} *HIS1 CaNMA111::13MYC-FLAG*	In this study
pJI434	*CEN amp*^{r} *URA3 nma111::hph-CaURA3-hph::nma111*	In this study
pJI435	*CEN amp*^{r} *URA3 nma111::hisG-CaURA3-hisG::nma111*	In this study
pJI436	*CEN amp*^{r} *URA3 ybh3::hph-CaURA3-hph::ybh3*	In this study
pJI437	*CEN amp*^{r} *URA3 ybh3::hisG-CaURA3-hisG::ybh3*	In this study

Table S2. Primers used in this study

**Primer name**	**Sequence**
DHH1_clon_F	CGGGATCCCAACAATATGACGGATACTAATTGG
DHH1_clon_R	CGACGCGTCGATATTGTTGAGCAGGATTTTGTGC
BIR1_clon_F	CGGGATCCCCAGTCAATGGCAAATATCAACC
BIR1_clon_R	CGACGCGTCGATCTTCTGTGGGCATCTCTTC
PAT1_clon_F	ATCGGCGGCCGCCATGTCATTTTTTGGGTTTGATCC
PAT1_clon_R	CGACGCGTCGTTGTTGAAGTTCTTTAATTTCAGTTTC
NMA111_clon_F	CGGGATCCCATCAGCATGTCAAGATATGG
NMA111_clon_R2	CGACGCGTCGTTCATTGTATTTTTTCTCCATCCAAG
YBH3_F	CGGGATCCCCTGATGTCTTCATATACTGAATTGCC
YBH3_R	CGACGCGTCGATCATCACGATTATTATTTAAG

1. Wilson, R.B., Davis, D. & Mitchell, A.P. Rapid hypothesis testing with *Candida albicans* through gene disruption with short homology regions. *J Bacteriol* **181**, 1868-1874 (1999).
2. Cao, F., Lane, S., Raniga, P.P., Lu, Y., Zhou, Z., Ramon, K., Chen, J. & Liu, H. The Flo8 transcription factor is essential for hyphal development and virulence in *Candida albicans*. *Mol Biol Cell* **17**, 295-307 (2006).
3. Stoldt, V. R., Sonneborn, A., Leuker, C. E. & Ernst, J. F. Efg1p, an essential regulator of morphogenesis of the human pathogen *Candida albicans*, is a member of a conserved class of bHLH proteins regulating morphogenetic processes in fungi. *EMBO J* **16,** 1982-1991 (1997).
