# Supplementary material for: Roles of the pro-apoptotic factors CaNma111 and CaYbh3 in apoptosis and virulence of Candida albicans
Source: Sci Rep. 2022 May 9;12:7574. doi: 10.1038/s41598-022-11682-y (PMC9085738; doi:10.1038/s41598-022-11682-y)
Supplement: Supplementary file 2 — Supplementary Information 2. [file 41598_2022_11682_MOESM2_ESM.pdf]

Roles of the pro-apoptotic factors CaNma111 and CaYbh3 in apoptosis and virulence of *Candida albicans*

Minsik Nam[1,2], Se Hyeon Kim[1,2], Jeong-Hoon Jeong[1], Su Young Kim[1],

*and Jinmi Kim[1]**

Supplementary Figure S1

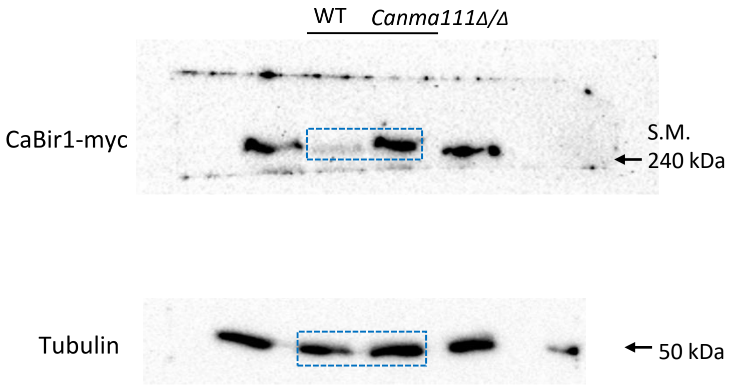

Supplementary Figure S1. Original gel images used to assemble Figure 4G.

Based on author guidelines, we provide the original immunoblots, which were used to assemble Figure 4G. The blots were cut prior to hybridization with anti-myc or anti-tubulin antibodies. We repeatedly observed a very faint protein band of CaBir1-myc in wild-type cells (see Supplementary Figure S2). For this reason, we are forced to overexpose the blot to enable this band to be compared with that of the mutant cells.

Supplementary Figure S2

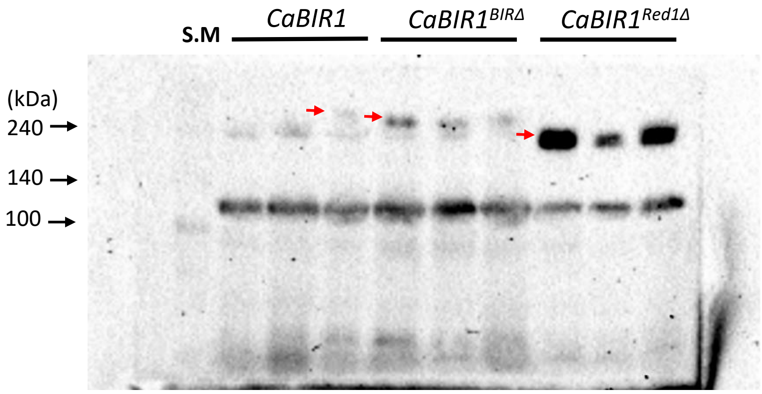

Supplementary Figure S2. Detection of the CaBir1-myc protein band. The pPR671-derived *ACT1-CaBIR1-MYC*, *ACT1-CaBIR1*$^{BIR\Delta}$*-MYC,* or *ACT1-CaBIR1*$^{Red1\Delta}$*-MYC* construct was chromosomally integrated in the wild-type BWP17 strain. Western blotting was conducted using anti-myc antibody. A faint band of the wild-type CaBir1-myc was detected above the size marker of 240 kDa. The deletion mutation of BIR domain (579 bp deletion) or Red1 domain (1,107 bp deletion) of CaBir1 showed a protein band lower than the wild-type band. Construction of these deletion mutations were described in our previous report[1].

1. Jeong, J. H., Kim, S. H. & Kim, J. CaBir1 functions as an inhibitor-of-apoptosis and affects caspase-like activitiy in *Candida albicans*. *Fungal Genet Biol* **154**, 103600, doi:10.1016/j.fgb.2021.103600 (2021).
